# Supplementary material for: Accurate prediction of protein assembly structure by combining AlphaFold and symmetrical docking
Source: Nat Commun. 2023 Dec 13;14:8283. doi: 10.1038/s41467-023-43681-6 (PMC10719378; doi:10.1038/s41467-023-43681-6)
Supplement: Supplementary file 1 — Supplementary Information [file 41467_2023_43681_MOESM1_ESM.docx]

**Supplementary Information**

**Accurate prediction of protein assembly structure by combining AlphaFold and symmetrical docking**

Mads Jeppesen^1^ and Ingemar André^1^*


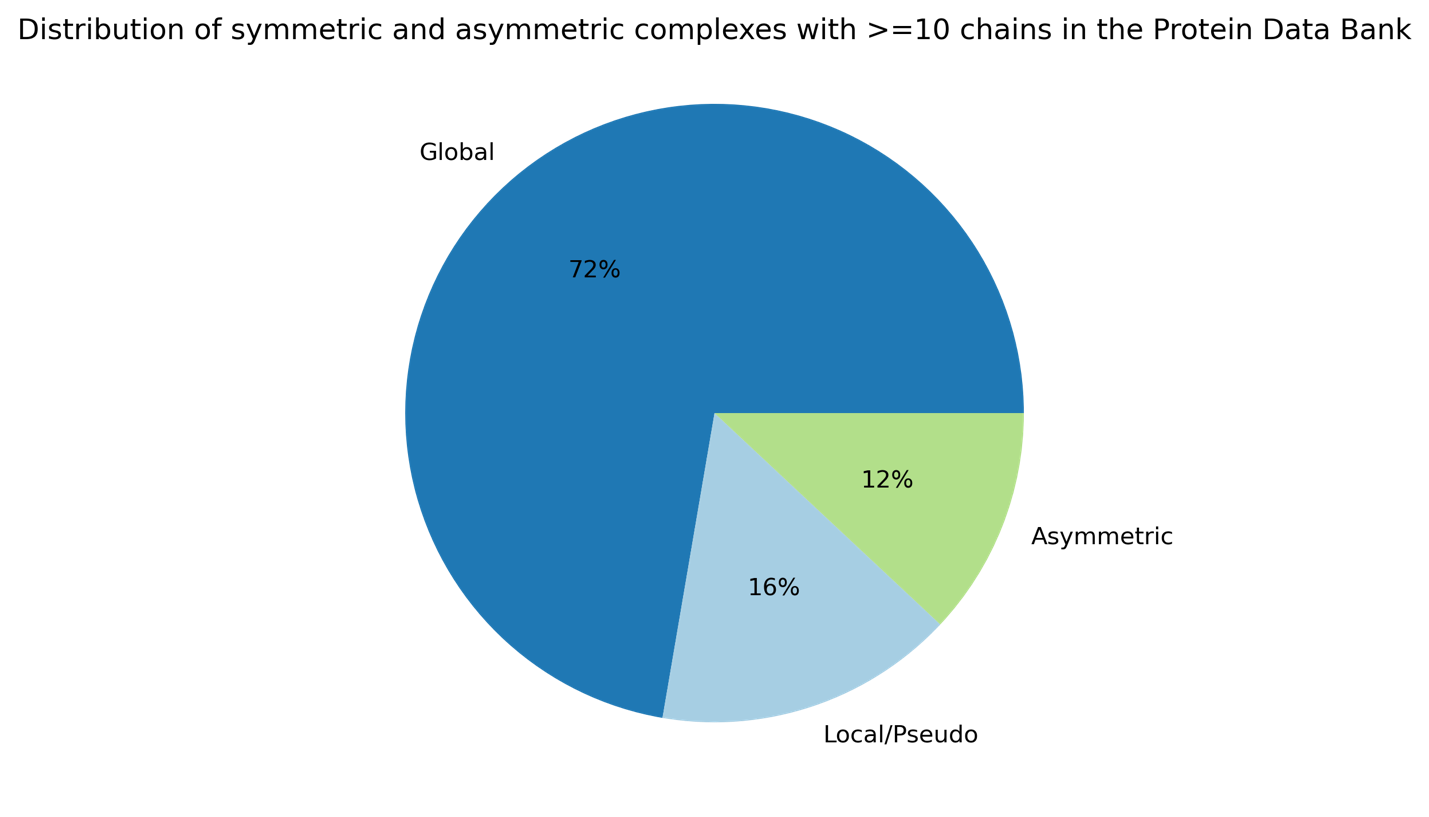


Figure S1**: Distribution of symmetric/asymmetric complexes with more than 10 chains in the Protein Data Bank**. The Protein Data Bank^1^ was culled of all PDBs containing 10 chains or more and containing no nucleic acids. These were then reduced by 30% sequence identity to a total number of 2062 individual PDBs. Source data are provided in the Source Data file.


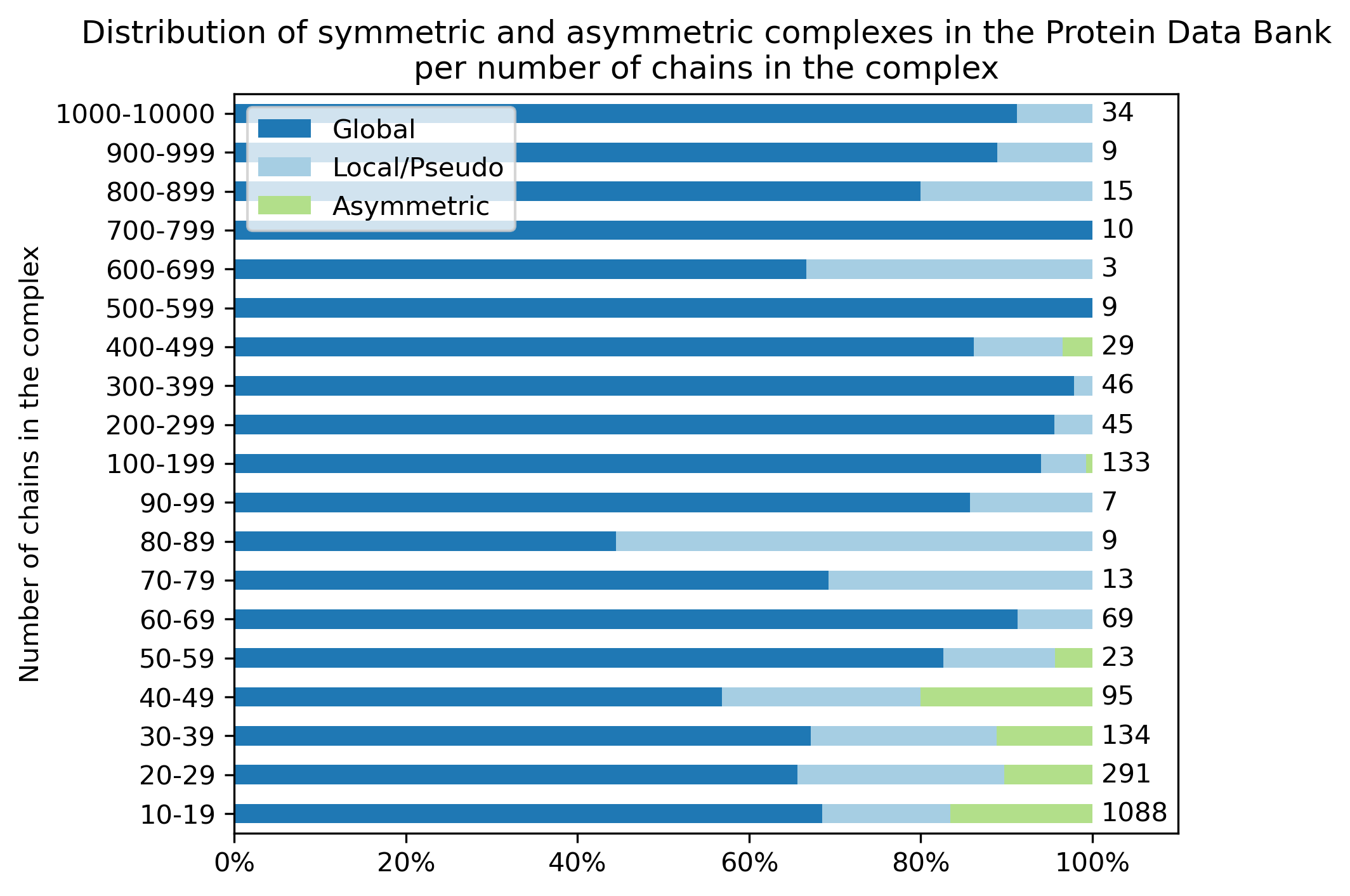


Figure S2**: Distribution of symmetric/asymmetric complexes in the Protein Data Bank per number of chains in the complex.** PDBs were culled as in S1 and binned from the number of chains in their complex (left y-label). The total amount in each bin is shown in the right y-label. Source data are provided in the Source Data file.


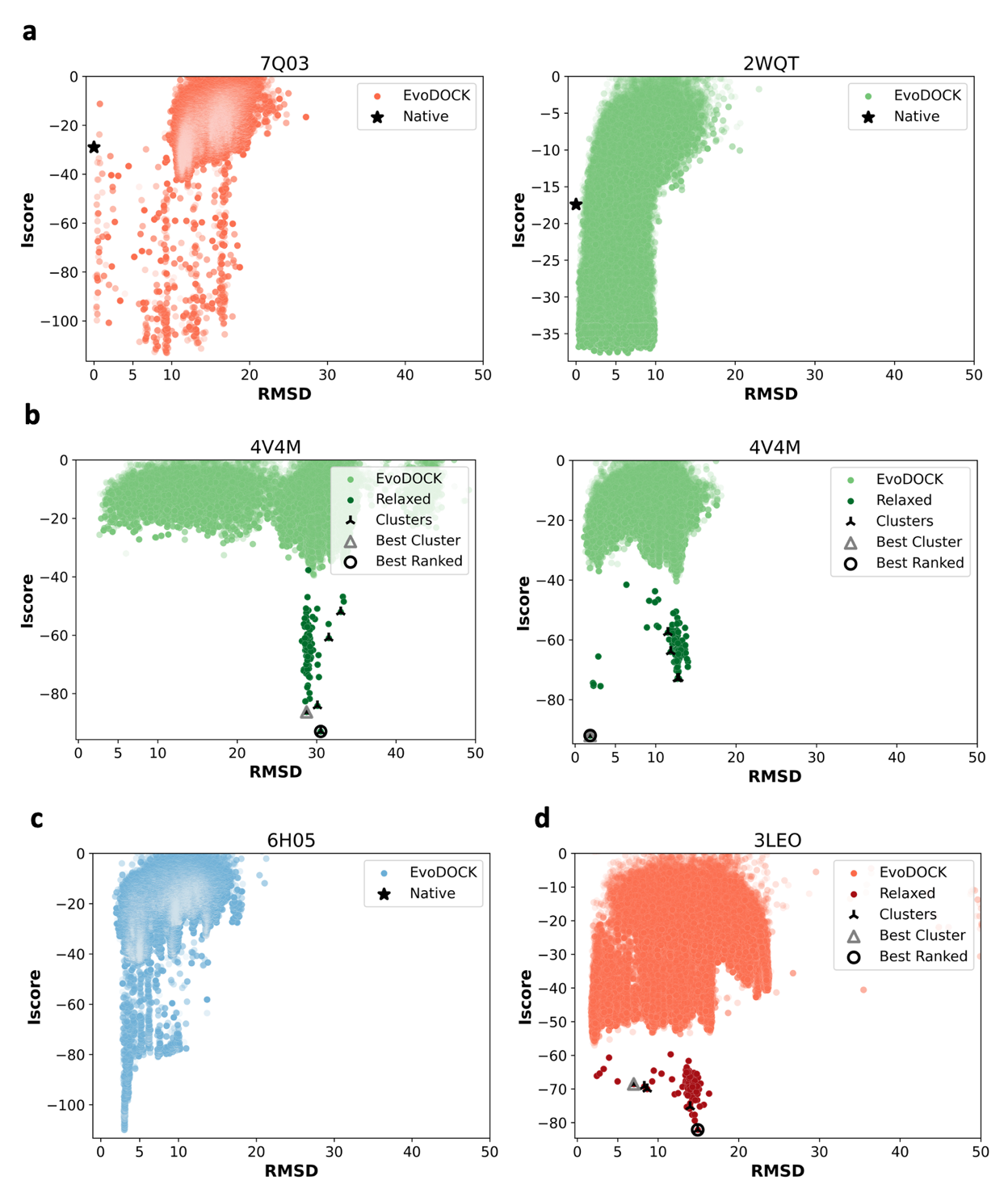


Figure S3: **Classification of failures to find near-native states.** **a:** 7Q03 (left) and 2WQT (right) are local recapitulation runs. EvoDOCK can sample the near-native state, but the energy function is not able to attribute the near-native state to the lowest energy. We therefore attribute the failure of EvoDOCK to find the near-native state to issues with Rosetta’s energy function. **b:**  In 4V4M (left), both docking orientations are allowed, while in 4V4M (right), only the correct oligomer orientation is allowed. This shows an example where the method is not able to pick out the right docking orientation when the combined docking approach is used. **c**: Local recapitulation simulation of 6H05. EvoDOCK fails to find the near-native state (at least below 2.5Å). Given the method has the correct backbone, the failure of the method to find the near-native state can be attributed to a sampling issue. **d:** Global assembly of 3LEO using only the correct orientation. The lowest energy model in the EvoDOCK simulation is a near-native state. When the clustered models are subjugated to energy refinement (Rosetta symmetric FastRelax), the energetically best models are identified as low non-native conformations. This case is attributed to problems in energy refinement rather than sampling in EvoDOCK. Source data are provided in the Source Data file*.*


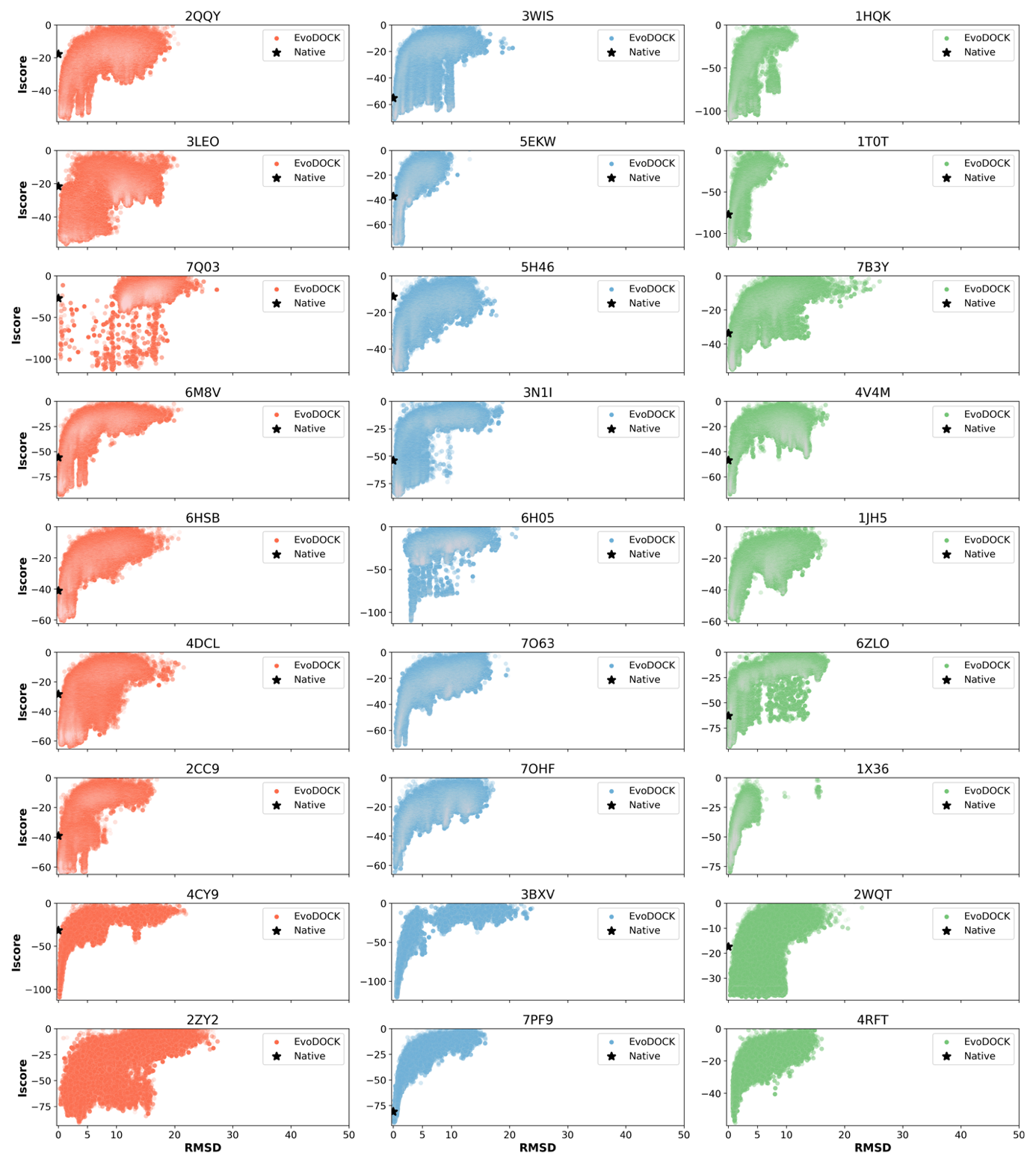


Figure S4: **Energy landscapes for local recapitulation docking.** Interface score and RMSD (Å) of subsystem relative to the known native structure (calculated as a subsystem). Energy values for the native capsid (symmetrically reconstructed) are illustrated with a star. If the native structure is not present in the plot, it means that the energy of it is above 0. This is the case for the following models: 2ZY2, 6H05, 7O63, 7OHF, 3BXV, 1HQK, 1JH5, 1X36, and 4RFT. Source data are provided in the Source Data file.


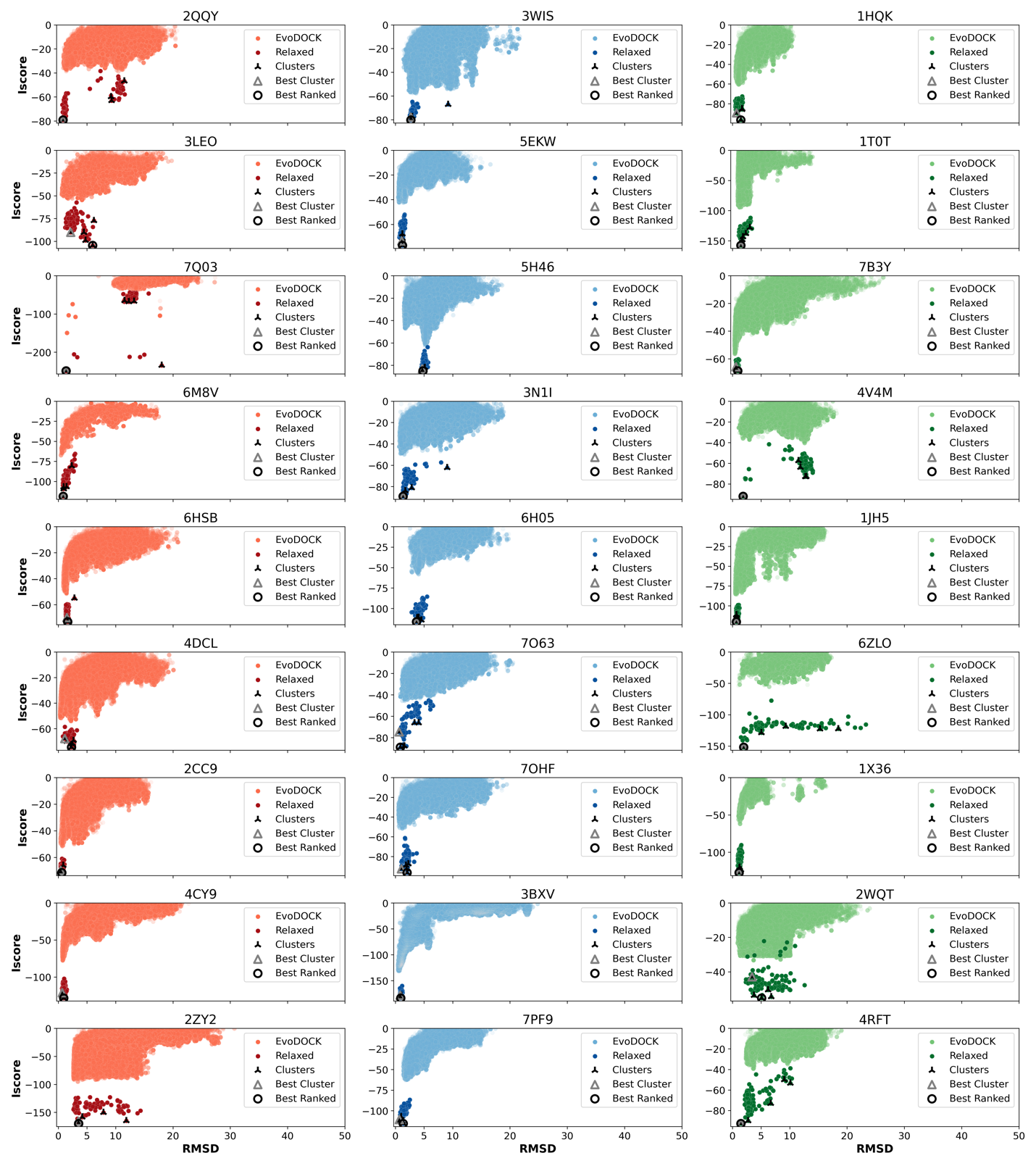


Figure S5: **Energy landscapes for local assembly docking.** Interface score and RMSD (Å) of subsystem relative to the known native structure (calculated as a subsystem). Models before and after energy refinement (relaxed) are illustrated, as well as the 5 selected clusters, the best cluster, and the energetically best model (best ranked). Source data are provided in the Source Data file.


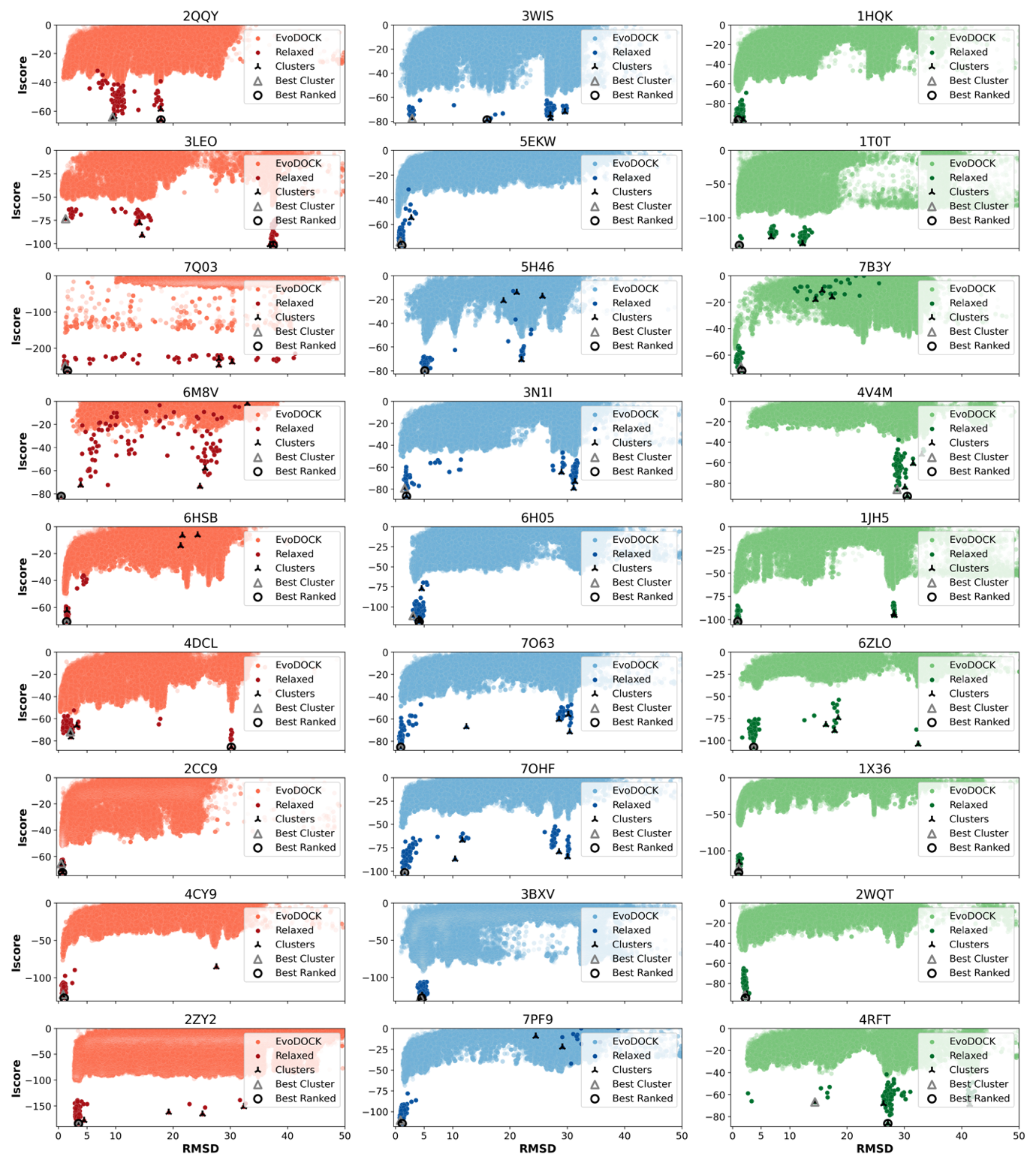


Figure S6**:** **Energy landscapes for global assembly docking.** Interface score and RMSD (Å) of subsystem relative to the known native structure (calculated as a subsystem). Models before and after energy refinement (relaxed) are illustrated, as well as the 5 selected clusters, the best cluster, and the energetically best model (best ranked). Source data are provided in the Source Data file.


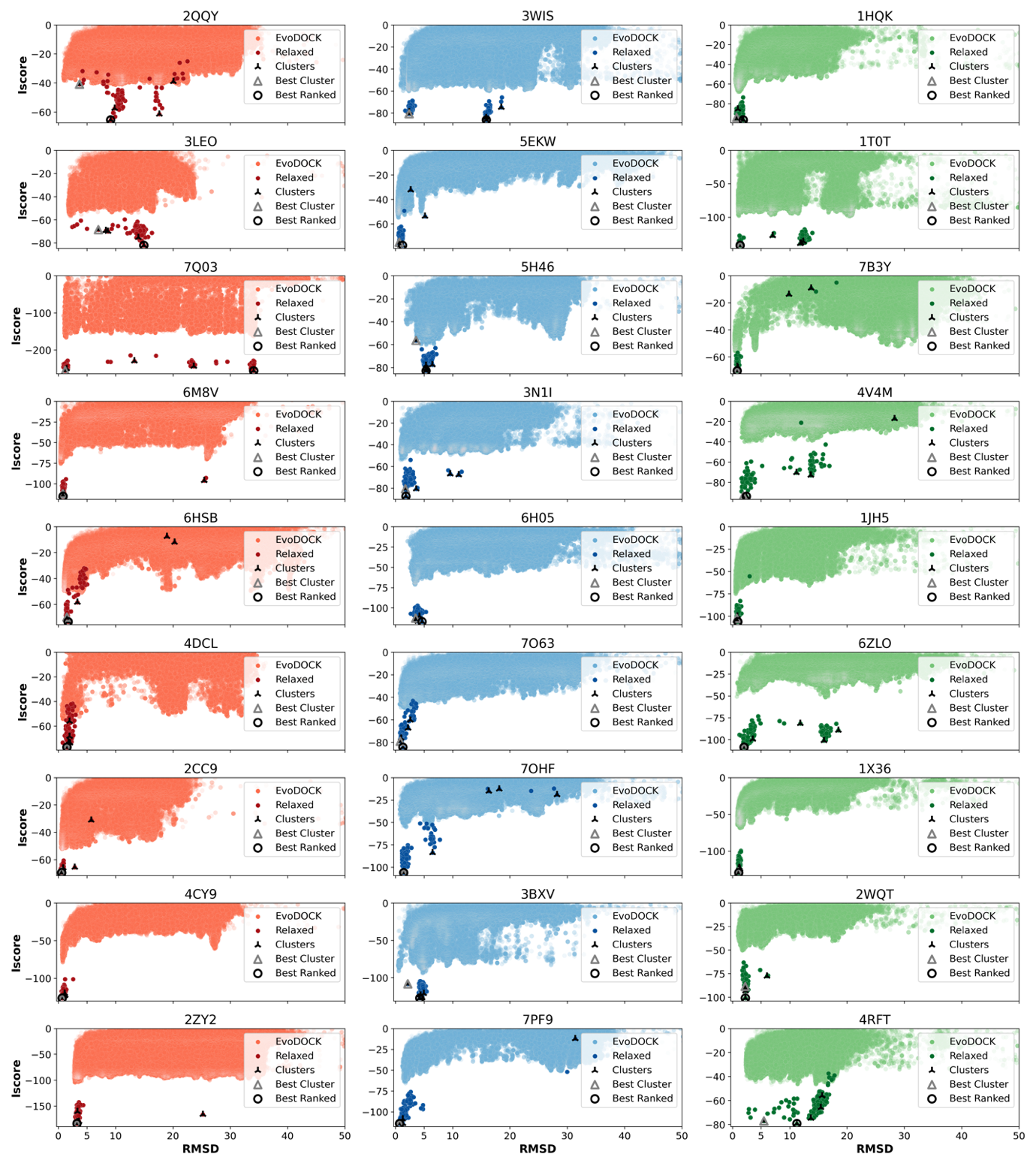


Figure S7**:** **Energy landscapes for global assembly docking assuming the correct orientation.** Interface score and RMSD (Å) of subsystem relative to the known native structure (calculated as a subsystem). Models before and after energy refinement (relaxed) are illustrated, as well as the 5 selected clusters, the best cluster, and the energetically best model (best ranked). Source data are provided in the Source Data file.


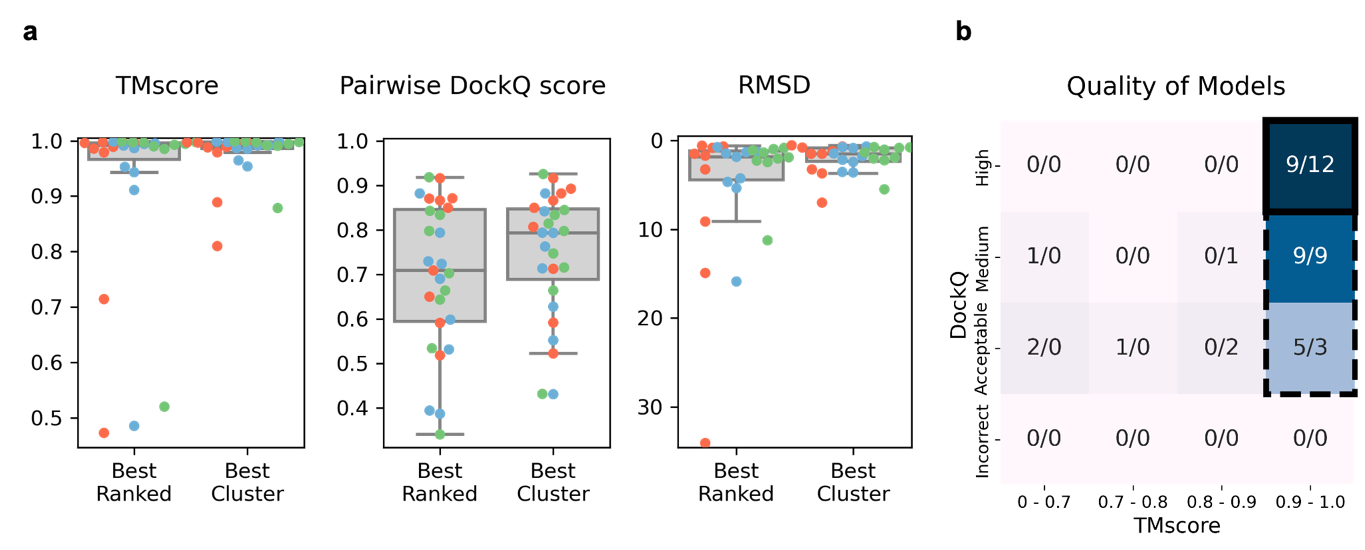


Figure S8: **Summary results for global assembly docking assuming the correct orientation. a:** TM-score (n=27), Pairwise DockQ score (n=27), and RMSD (n=27) for all benchmark structures (T=red, O=blue, I=green). **b:** Classification of the results using TM-score and DockQ. The outer dashed line delineates successfully predicted structures, and the inner black box shows highly accurate structures.


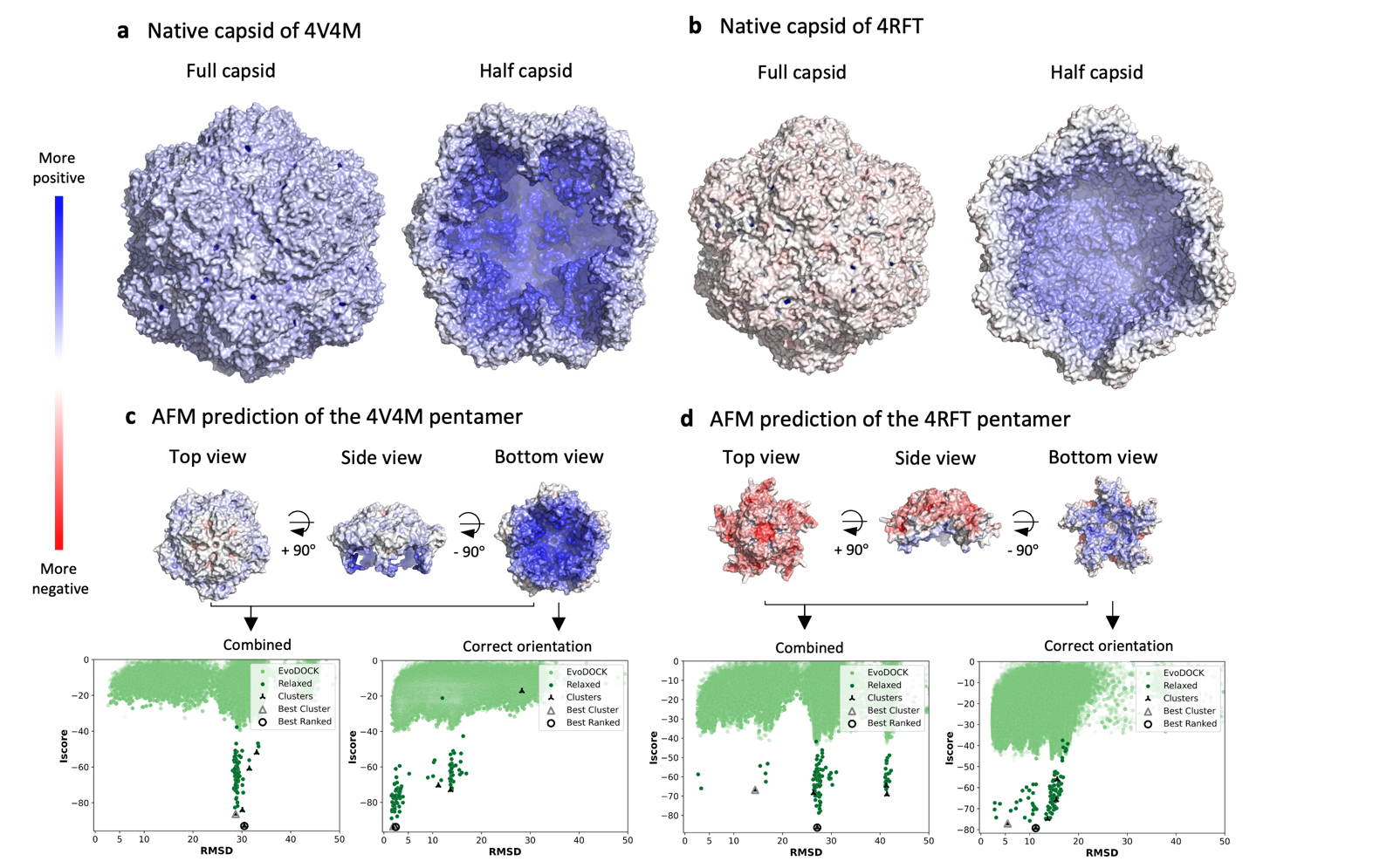


Figure S9: **Selecting docking orientation based on charge distribution can improve results.** Viral capsids often display positive charges on the interior to help facilitate the encapsulation of negatively charged nucleic acids^2^**. a:** Charge distribution of the native capsid of the PDB 4V4M showing a view of the full (left) and half (right) capsid. Notice the charge is more positive on the inside. Charge distributions were calculated with APBS^3^. **b**: Same as in A but for the native capsid of the PDB 4RFT. **c.** The topmost part of the figure shows three views of the pentameric prediction of AFM (symmetrized by Rosetta) distinguished by a 180$^{\circ}$ rotation between them. Notice the bottom of the pentamer is more positively charged than the top. The bottom part of the figure shows two RMSD plots where either the top and bottom orientation of the pentamer has been docked (left) in combination or only the bottom orientation (right). Selecting the orientation so that the most positively charged part of the protein is pointing inwards (bottom/correct orientation) can significantly improve the results. **d**. Same as in C but for the PDB 4RFT. Source data for Figure S9c-d are provided in the Source Data file.


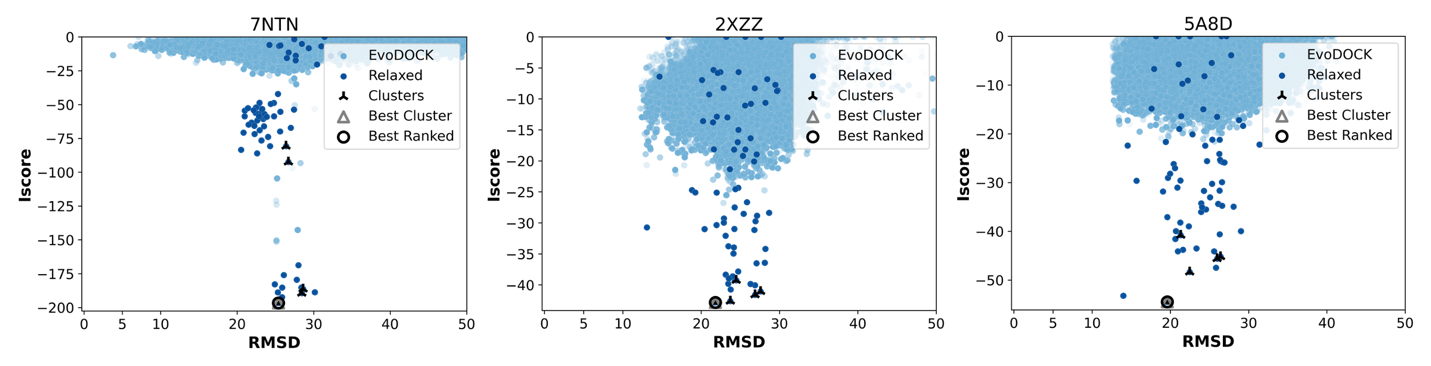


Figure S10: **Failure of the method to produce near-native models if not guided by AFM oligomer spatial information.** The Models (7NTN, 2XZZ, 5A8D) were selected based on the criteria that AFM produced a model with ipTM+pTM < 0.9 and average pLDDT >= 90. The results of the RMSD plots highlight the difficulty of the model on its own without using AFM to produce starting positions to find the near-native state. Source data are provided in the Source Data file*.*


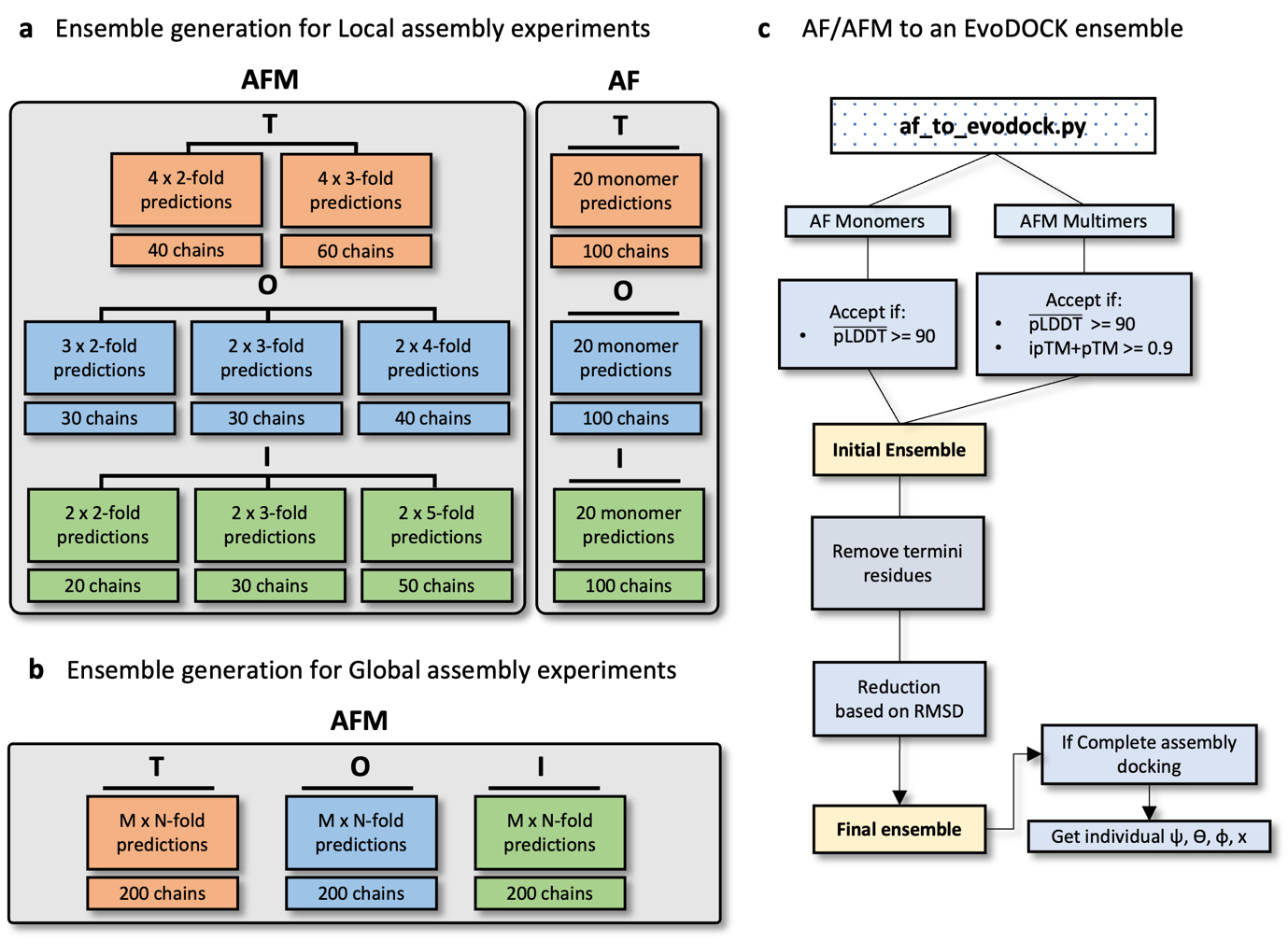


Figure S11: **Ensemble generation methodology. a:** Ensemble generation strategy for the local assembly experiments. Each box for AFM (left) and AF (right) shows how many predictions for each cubic symmetry type were run and how many chains they produced in total. **b:** Ensemble generation for the global assembly experiments. For a given oligomeric prediction (N-fold), AFM is run M times to produce a total of 200 chains. For instance, for a 5-fold prediction, M is 40 as AFM produces 5 chains for each prediction. In the case of the 3-fold, more predictions were run, and finally, only 200 chains were selected. **c**: The internals of the af_to_evodock.py script that is run to generate an input ensemble for EvoDOCK from AF/AFM predictions. Boxes are described in the main text.

Table S1: **Benchmark set, utilized symmetry and homology to subunits in PDB.**

| **PDB** | **Symmetry** | **Local**  **symmetry types^1^** | **Global**  **symmetry type^2^** | **AFM training set homology^3^** |
| --- | --- | --- | --- | --- |
| **Benchmark PDBs** | | | | |
| 4DCL | T | 3 | 3 | 92% |
| 2CC9 | T | 3 | 3 | 39% |
| 3LEO | T | 3 | 3 | 100% |
| 2QQY | T | 3 | 2 | 27% |
| 7Q03 | T | 3 | 2 | 51% |
| 6M8V | T | 3 | 2 | 51% |
| 6HSB | T | 3 | 3 | 62% |
| 4CY9 | T | 3 | 2 | 25% |
| 2ZY2 | T | 3 | 2 | 21% |
| 3WIS | O | 4 | 3 | 24% |
| 5H46 | O | 4 | 2 | 31% |
| 5EKW | O | 4 | 3 | 24% |
| 3N1I | O | 4 | 4 | 100% |
| 6H05 | O | 4 | 3 | 33% |
| 7O63 | O | 4 | 2 | 60% |
| 7OHF | O | 4 | 2 | 51% |
| 3BXV | O | 4 | 2 | 21% |
| 7PF9 | O | 4 | 4 | 24% |
| 1HQK | I | 5 | 5 | 48% |
| 1T0T | I | 5 | 5 | 100% |
| 1X36 | I | 5 | 5 | 22% |
| 7B3Y | I | 5 | 3 | 58% |
| 4V4M | I | 5 | 5 | 22% |
| 1JH5 | I | 5 | 3 | 95% |
| 6ZLO | I | 5 | 3 | 27% |
| 2WQT | I | 5 | 5 | 22% |
| 4RFT | I | 5 | 5 | 23% |
| **PDBs selected with ipTM+pTM < 0.9 and pLDDT >= 90 from AFM predictions** | | | | |
| 2XZZ | O | Not modelled | 4 | 24% |
| 5A8D | O | Not modelled | 2 | 26% |
| 7NTN | O | Not modelled | 2 | 28% |

^1^Symmetry of AFM prediction used for local assembly. ^2^Symmetry of AFM prediction used for global assembly. ^3^Sequence identity to proteins in the PDB released before 2018-04-30.

Table S2: **Expected computational time to reach a high level of accuracy for EvoDOCK.**

|  | **Local assembly** | | | | | | **Global assembly** | | | | | |
| --- | --- | --- | --- | --- | --- | --- | --- | --- | --- | --- | --- | --- |
|  | **80%** | | **90%** | | **99%** | | **80%** | | **90%** | | **99%** | |
|  | **Runs** | **Time** | **Runs** | **Time** | **Runs** | **Time** | **Runs** | **Time** | **Runs** | **Time** | **Runs** | **Time** |
| **Mean** | 9 | 491 | 13 | 724 | 18 | 986 | 13 | 374 | 18 | 496 | 30 | 793 |
| **Median** | 1 | 42 | 1 | 48 | 2 | 90 | 2 | 54 | 3 | 60 | 10 | 301 |

Time is displayed in hours.

Table S3: **Metric values for the local assembly experiments**

| **PDB** | **TM-score** | | **Pairwise DockQ score** | | **RMSD** | |
| --- | --- | --- | --- | --- | --- | --- |
|  | Best Ranked | Best Cluster | Best Ranked | Best Cluster | Best Ranked | Best Cluster |
| 4DCL | 0.97 | 0.99 | 0.87 | 0.87 | 2.31 | 1.13 |
| 2CC9 | 1.00 | 1.00 | 0.86 | 0.88 | 0.61 | 0.43 |
| 3LEO | 0.93 | 0.97 | 0.57 | 0.74 | 6.00 | 2.17 |
| 2QQY | 0.96 | 0.96 | 0.86 | 0.86 | 0.83 | 0.83 |
| 7Q03* | 0.99 | 0.99 | 0.82 | 0.82 | 1.36 | 1.36 |
| 6M8V | 1.00 | 1.00 | 0.81 | 0.81 | 0.86 | 0.86 |
| 6HSB | 0.99 | 0.99 | 0.84 | 0.87 | 1.65 | 1.50 |
| 4CY9 | 1.00 | 1.00 | 0.86 | 0.90 | 0.95 | 0.69 |
| 2ZY2* | 0.97 | 0.98 | 0.59 | 0.60 | 3.55 | 3.52 |
| 3WIS | 0.98 | 0.98 | 0.57 | 0.60 | 2.71 | 2.70 |
| 5H46 | 0.93 | 0.93 | 0.55 | 0.56 | 4.82 | 4.71 |
| 5EKW | 0.99 | 1.00 | 0.69 | 0.86 | 1.25 | 1.12 |
| 3N1I | 0.99 | 0.99 | 0.63 | 0.73 | 1.36 | 1.36 |
| 6H05 | 0.96 | 0.96 | 0.40 | 0.43 | 3.69 | 3.69 |
| 7O63 | 1.00 | 1.00 | 0.73 | 0.73 | 0.88 | 0.66 |
| 7OHF | 0.98 | 1.00 | 0.66 | 0.84 | 2.05 | 1.06 |
| 3BXV* | 1.00 | 1.00 | 0.89 | 0.89 | 0.95 | 0.84 |
| 7PF9 | 0.99 | 1.00 | 0.80 | 0.88 | 1.38 | 0.69 |
| 1HQK | 0.99 | 1.00 | 0.70 | 0.83 | 1.47 | 0.72 |
| 1T0T | 1.00 | 1.00 | 0.79 | 0.92 | 1.48 | 1.48 |
| 1X36 | 1.00 | 1.00 | 0.82 | 0.82 | 1.17 | 1.17 |
| 7B3Y | 1.00 | 1.00 | 0.92 | 0.93 | 0.94 | 0.62 |
| 4V4M | 0.99 | 0.99 | 0.70 | 0.70 | 1.87 | 1.87 |
| 1JH5 | 1.00 | 1.00 | 0.91 | 0.91 | 0.69 | 0.69 |
| 6ZLO | 0.99 | 0.99 | 0.76 | 0.76 | 1.97 | 1.97 |
| 2WQT | 0.96 | 0.98 | 0.63 | 0.63 | 5.10 | 3.43 |
| 4RFT | 0.99 | 0.99 | 0.69 | 0.69 | 1.49 | 1.49 |

* Due to utilizing a subsystem some conformations for these PDBs did not match the subsystem of the experimental structures used to calculate the RMSD with. A mix between 4, 5 and all chains were used to calculate the RMSD.

Table S4: **All metric values for the global assembly experiments**

| **PDB** | **TM-score** | | **Pairwise DockQ score** | | **RMSD** | |
| --- | --- | --- | --- | --- | --- | --- |
|  | Best Ranked | Best Cluster | Best Ranked | Best Cluster | Best Ranked | Best Cluster |
| 4DCL | 0.20 | 0.98 | 0.65 | 0.80 | 30.18 | 2.11 |
| 2CC9 | 0.99 | 1.00 | 0.86 | 0.88 | 0.73 | 0.50 |
| 3LEO | 0.33 | 0.99 | 0.67 | 0.83 | 37.48 | 1.27 |
| 2QQY | 0.71 | 0.71 | 0.53 | 0.53 | 17.91 | 9.47 |
| 7Q03* | 0.99 | 1.00 | 0.79 | 0.84 | 1.59 | 1.19 |
| 6M8V | 1.00 | 1.00 | 0.91 | 0.91 | 0.50 | 0.50 |
| 6HSB | 0.99 | 0.99 | 0.85 | 0.85 | 1.46 | 1.46 |
| 4CY9 | 0.99 | 1.00 | 0.85 | 0.86 | 1.04 | 0.87 |
| 2ZY2* | 0.97 | 0.97 | 0.57 | 0.57 | 3.53 | 3.53 |
| 3WIS | 0.49 | 0.98 | 0.39 | 0.55 | 16.00 | 2.94 |
| 5H46 | 0.92 | 0.92 | 0.55 | 0.55 | 5.14 | 5.14 |
| 5EKW | 1.00 | 1.00 | 0.71 | 0.78 | 1.15 | 0.87 |
| 3N1I | 0.98 | 0.99 | 0.68 | 0.68 | 1.95 | 1.63 |
| 6H05 | 0.95 | 0.97 | 0.41 | 0.50 | 4.15 | 3.10 |
| 7O63 | 1.00 | 1.00 | 0.82 | 0.82 | 0.94 | 0.94 |
| 7OHF | 0.99 | 0.99 | 0.74 | 0.74 | 1.63 | 1.63 |
| 3BXV* | 0.94 | 0.95 | 0.57 | 0.61 | 4.61 | 4.36 |
| 7PF9 | 1.00 | 1.00 | 0.82 | 0.86 | 1.21 | 0.90 |
| 1HQK | 1.00 | 1.00 | 0.76 | 0.82 | 1.06 | 0.75 |
| 1T0T | 1.00 | 1.00 | 0.80 | 0.80 | 1.21 | 1.21 |
| 1X36 | 1.00 | 1.00 | 0.83 | 0.84 | 1.10 | 1.09 |
| 7B3Y | 0.99 | 0.99 | 0.89 | 0.91 | 1.64 | 1.48 |
| 4V4M | 0.44 | 0.48 | 0.05 | 0.13 | 30.51 | 28.74 |
| 1JH5 | 1.00 | 1.00 | 0.82 | 0.84 | 0.94 | 0.88 |
| 6ZLO | 0.98 | 0.98 | 0.44 | 0.48 | 3.72 | 3.72 |
| 2WQT | 0.99 | 0.99 | 0.72 | 0.72 | 2.29 | 2.19 |
| 4RFT | 0.44 | 0.50 | 0.34 | 0.34 | 27.12 | 14.40 |

* Due to utilizing a subsystem some conformations for these PDBs did not match the subsystem of the experimental structures used to calculate the RMSD with. A mix between 4, 5 and all chains were used to calculate the RMSD.

Table S5: **All metric values for the single direction global assembly experiments**

| **PDB** | **TM-score** | | **Pairwise DockQ score** | | **RMSD** | |
| --- | --- | --- | --- | --- | --- | --- |
|  | Best Ranked | Best Cluster | Best Ranked | Best Cluster | Best Ranked | Best Cluster |
| 4DCL | 0.99 | 0.99 | 0.71 | 0.81 | 1.50 | 1.50 |
| 2CC9 | 1.00 | 1.00 | 0.87 | 0.88 | 0.59 | 0.54 |
| 3LEO | 0.47 | 0.81 | 0.65 | 0.71 | 14.92 | 7.00 |
| 2QQY | 0.71 | 0.89 | 0.52 | 0.52 | 9.13 | 3.70 |
| 7Q03* | 1.00 | 1.00 | 0.87 | 0.87 | 34.08 | 1.19 |
| 6M8V | 1.00 | 1.00 | 0.87 | 0.89 | 0.83 | 0.80 |
| 6HSB | 0.99 | 0.99 | 0.85 | 0.85 | 1.71 | 1.50 |
| 4CY9 | 1.00 | 1.00 | 0.92 | 0.92 | 0.67 | 0.67 |
| 2ZY2* | 0.98 | 0.98 | 0.59 | 0.59 | 3.25 | 3.25 |
| 3WIS | 0.49 | 0.99 | 0.39 | 0.63 | 15.88 | 2.42 |
| 5H46 | 0.91 | 0.95 | 0.53 | 0.55 | 5.36 | 3.61 |
| 5EKW | 1.00 | 1.00 | 0.73 | 0.79 | 1.26 | 0.67 |
| 3N1I | 0.99 | 0.99 | 0.69 | 0.71 | 1.84 | 1.76 |
| 6H05 | 0.94 | 0.96 | 0.39 | 0.43 | 4.66 | 3.54 |
| 7O63 | 0.99 | 1.00 | 0.72 | 0.84 | 1.32 | 0.85 |
| 7OHF | 0.99 | 0.99 | 0.79 | 0.79 | 1.46 | 1.46 |
| 3BXV* | 0.95 | 0.99 | 0.60 | 0.76 | 4.27 | 2.18 |
| 7PF9 | 1.00 | 1.00 | 0.88 | 0.88 | 0.74 | 0.74 |
| 1HQK | 0.99 | 1.00 | 0.64 | 0.81 | 1.90 | 0.73 |
| 1T0T | 1.00 | 1.00 | 0.80 | 0.80 | 1.35 | 1.35 |
| 1X36 | 1.00 | 1.00 | 0.83 | 0.83 | 1.11 | 1.03 |
| 7B3Y | 1.00 | 1.00 | 0.92 | 0.93 | 0.84 | 0.84 |
| 4V4M | 0.99 | 0.99 | 0.53 | 0.75 | 2.44 | 1.92 |
| 1JH5 | 1.00 | 1.00 | 0.84 | 0.84 | 0.96 | 0.77 |
| 6ZLO | 0.99 | 0.99 | 0.66 | 0.66 | 2.04 | 2.04 |
| 2WQT | 0.99 | 0.99 | 0.70 | 0.72 | 2.27 | 2.24 |
| 4RFT | 0.52 | 0.88 | 0.34 | 0.43 | 11.25 | 5.50 |

* Due to utilizing a subsystem some conformations for these PDBs did not match the subsystem of the experimental structures used to calculate the RMSD with. A mix between 4, 5 and all chains were used to calculate the RMSD.

**Supplementary References**

1. Berman HM*, et al.* The Protein Data Bank. *Nucleic Acids Res* **28**, 235-242 (2000).

2. Perlmutter JD, Hagan MF. Mechanisms of Virus Assembly. *Annu Rev Phys Chem* **66**, 217-239 (2015).

3. Jurrus E*, et al.* Improvements to the APBS biomolecular solvation software suite. *Protein Sci* **27**, 112-128 (2018).
