# Supplementary material for: Accurate prediction of protein assembly structure by combining AlphaFold and symmetrical docking
Source: Nat Commun. 2023 Dec 13;14:8283. doi: 10.1038/s41467-023-43681-6 (PMC10719378; doi:10.1038/s41467-023-43681-6)
Supplement: Supplementary file 3 — Reporting Summary [file 41467_2023_43681_MOESM3_ESM.pdf]

nature portfolio

Corresponding author(s): Ingemar André

Last updated by author(s): Nov 10, 2023

Reporting Summary

Nature Portfolio wishes to improve the reproducibility of the work that we publish. This form provides structure for consistency and transparency in reporting. For further information on Nature Portfolio policies, see our Editorial Policies and the Editorial Policy Checklist.

Statistics

For all statistical analyses, confirm that the following items are present in the figure legend, table legend, main text, or Methods section.

n/a	Confirmed	
☐	☒	The exact sample size (*n*) for each experimental group/condition, given as a discrete number and unit of measurement
☐	☒	A statement on whether measurements were taken from distinct samples or whether the same sample was measured repeatedly
☒	☐	The statistical test(s) used AND whether they are one- or two-sided<br>*Only common tests should be described solely by name; describe more complex techniques in the Methods section.*
☒	☐	A description of all covariates tested
☒	☐	A description of any assumptions or corrections, such as tests of normality and adjustment for multiple comparisons
☐	☒	A full description of the statistical parameters including central tendency (e.g. means) or other basic estimates (e.g. regression coefficient) AND variation (e.g. standard deviation) or associated estimates of uncertainty (e.g. confidence intervals)
☒	☐	For null hypothesis testing, the test statistic (e.g. *F*, *t*, *r*) with confidence intervals, effect sizes, degrees of freedom and *P* value noted<br>*Give P values as exact values whenever suitable.*
☒	☐	For Bayesian analysis, information on the choice of priors and Markov chain Monte Carlo settings
☒	☐	For hierarchical and complex designs, identification of the appropriate level for tests and full reporting of outcomes
☒	☐	Estimates of effect sizes (e.g. Cohen's *d*, Pearson's *r*), indicating how they were calculated

Our web collection on statistics for biologists contains articles on many of the points above.

Software and code

Policy information about availability of computer code

Data collection	Custom bash (version=5.0.17) and python (version=3.7) scripts were used collect the data as carried out in this study. The following libraries for python were used: pandas=1.3.5, biopython=1.79, numpy=1.21.2, pyrosetta=(git commit: 676ce5806b2). Code for Symmetric EvoDock is found at https://github.com/Andre-lab/evodock.
Data analysis	Custom python (=3.7) scripts were used analyze the data as carried out in this study. The following libraries were used: pandas=1.3.5, biopython=1.79, numpy=1.21.2, pyrosetta=(git commit: 676ce5806b2), seaborn= 0.12.2, matplotlib=3.5.1.

For manuscripts utilizing custom algorithms or software that are central to the research but not yet described in published literature, software must be made available to editors and reviewers. We strongly encourage code deposition in a community repository (e.g. GitHub). See the Nature Portfolio guidelines for submitting code & software for further information.

Data

Policy information about availability of data

All manuscripts must include a data availability statement. This statement should provide the following information, where applicable:

- Accession codes, unique identifiers, or web links for publicly available datasets
- A description of any restrictions on data availability
- For clinical datasets or third party data, please ensure that the statement adheres to our policy

Data collected in the study is available at https://github.com/Andre-lab/af_cubic_docking

Research involving human participants, their data, or biological material

Policy information about studies with human participants or human data. See also policy information about sex, gender (identity/presentation), and sexual orientation and race, ethnicity and racism.

Reporting on sex and gender	N/A
Reporting on race, ethnicity, or other socially relevant groupings	N/A
Population characteristics	N/A
Recruitment	N/A
Ethics oversight	N/A

Note that full information on the approval of the study protocol must also be provided in the manuscript.

Field-specific reporting

Please select the one below that is the best fit for your research. If you are not sure, read the appropriate sections before making your selection.

[x] Life sciences [ ] Behavioural & social sciences [ ] Ecological, evolutionary & environmental sciences

For a reference copy of the document with all sections, see nature.com/documents/nr-reporting-summary-flat.pdf

Life sciences study design

All studies must disclose on these points even when the disclosure is negative.

Sample size	The benchmark set was selected to provide 9 cases for each category of symmetry studied in this work. This number is a tradeoff between statistics to draw conclusions about average performance of the method and computational time. The dataset was selected to contain systems with a range of homology values compared to models that may have been used for training of the AlphaFold-Multimer method. With 27 systems, the data is sufficient to calculate a average values of structural metrics with some confidence, but to accurately characterize the statistical distribution of values more data would likely be needed.
Data exclusions	Structures that failed to be predicted by AlphaFold 2 and AlphaFold-multimer for various reasons were not continued.
Replication	The reported result was the result of a single run of the structural benchmark. However, each protein system is simulated 100 times independently and the data collected for the final result, so there is repetition in the simulation data. There was a significant oversampling for each system and with this data we can investigate the effect of random sampling, which we do when we evaluate time complexity of the method.<br><br>In addition, several variations of the benchmark was run, including with and without knowledge about the orientation of oligomers. These results were highly consistent. We also ran several types of benchmarks on the same system. In total more than 100 benchmark simulations were carried out and reported in the study.
Randomization	Samples were not allocated in groups and compared apart from the study of time complexity of the simulation, in which random sets of independent EvoDOCK runs were sampled.
Blinding	Blinding was not possible. In the structure prediction field there is the possiblity to participate in the CASP competition to receive blind prediction cases. This competation run every two years. This years competition did not include any cubic symmetry systems that could have been used for blind prediction and to the best of our knowledge these types of systems have not been part of CASP prior to this. However, we have tried to reduce information from known structures by the selection of templates for modeling and only including inclusion of cases that were deposited after AlphaFold and AlphaFold-Multimer were trained.

Reporting for specific materials, systems and methods

We require information from authors about some types of materials, experimental systems and methods used in many studies. Here, indicate whether each material, system or method listed is relevant to your study. If you are not sure if a list item applies to your research, read the appropriate section before selecting a response.

Materials & experimental systems

n/a	Involved in the study	
☒	☐	Antibodies
☒	☐	Eukaryotic cell lines
☒	☐	Palaeontology and archaeology
☒	☐	Animals and other organisms
☒	☐	Clinical data
☒	☐	Dual use research of concern
☒	☐	Plants

Methods

n/a	Involved in the study	
☒	☐	ChIP-seq
☒	☐	Flow cytometry
☒	☐	MRI-based neuroimaging
